# Natural killer cells suppress cancer metastasis by eliminating circulating cancer cells

**DOI:** 10.3389/fimmu.2022.1098445

**Published:** 2023-01-17

**Authors:** Maulik Vyas, Marta Requesens, Thao H. Nguyen, Domitille Peigney, Marjan Azin, Shadmehr Demehri

**Affiliations:** Department of Dermatology and Center for Cancer Research, Massachusetts General Hospital and Harvard Medical School, Boston, MA, United States

**Keywords:** NK cells, metastatic melanoma, T cells, helper function, cancer immunology

## Abstract

Despite significant advances in cancer treatment, the metastatic spread of malignant cells to distant organs remains a major cause of cancer-related deaths. Natural killer (NK) cells play a crucial role in controlling tumor metastasis; however, the dynamics of NK cell-mediated clearance of metastatic tumors are not entirely understood. Herein, we demonstrate the cooperative role of NK and T cells in the surveillance of melanoma metastasis. We found that NK cells effectively limited the pulmonary seeding of B16 melanoma cells, while T cells played a primary role in restricting metastatic foci growth in the lungs. Although the metastatic foci in the lungs at the endpoint were largely devoid of NK cells, they played a prominent role in promoting T cell recruitment into the metastatic foci. Our data suggested that the most productive interaction between NK cells and metastatic cancer cells occurred when cancer cells were in circulation. Modifying the route of administration so that intravenously injected melanoma cells bypass the first liver passage resulted in significantly more melanoma metastasis to the lung. This finding indicated the liver as a prominent site where NK cells cleared melanoma cells to regulate their seeding in the lungs. Consistent with this notion, the liver and the lungs of the tumor-bearing mice showed dominance of NK and T cell activation, respectively. Thus, NK cells and T cells control pulmonary metastasis of melanoma cells by distinct mechanisms where NK cells play a critical function in shaping T cell-mediated *in situ* control of lung-seeded cancer cells. A precise understanding of the cooperative role of NK and T cells in controlling tumor metastasis will enable the development of the next generation of cancer immunotherapies.

## Introduction

There have been significant advances in the past decade to further our understanding of how cancers arise and progress and how the immune system impacts the course of the disease. Despite these colossal efforts leading to the unprecedented decline in the overall cancer death rate in the last ten years, cancer remains the second leading cause of death worldwide (1). Most cancer-related deaths occur due to metastasis (2). Since the publication of a seminal “seed and soil” theory in 1889 to hypothesize how a particular tumor may spread to specific distant organs (3), a growing number of studies have identified the tumor-intrinsic mechanisms regulating various stages of cancer metastasis (4, 5). It is increasingly becoming evident that the immune system plays an important role in controlling the dissemination of cancer cells and their subsequent growth in distant organs (6, 7). To date, natural killer (NK) and T cells are the most utilized immune effector cells to potentiate this effect using various cancer immunotherapeutic approaches.

NK cells are innate immune cells with the potent ability to rapidly kill infected and malignantly transformed cells (8). NK cell activation is regulated by a variety of germline-encoded activating and inhibitory receptors to prevent the non-specific destruction of normal tissue while enabling the clearance of target cells (8). In contrast, T cells are part of adaptive immunity and require co-stimulation to mount a robust immune response against an antigen presented by major histocompatibility complexes (MHC), which serves as the primary stimulatory signal (9). The important role of T cells in cancer surveillance is demonstrated by several cancer immunotherapies including immune checkpoint blockade, T cell engagers and CAR T cell therapies which exploit T cell-directed immune responses to significantly control tumor burden in hematological and solid cancer patients (9). On the other hand, while NK cell-mediated surveillance of primary solid tumors remains a matter of debate, their role in controlling hematological cancers and limiting the metastatic spread of the primary tumors is well established (10–14). NK cell effectiveness in solid cancers is severely hindered by their inadequate infiltration and inhibition by immunosuppressive tumor microenvironment (TME) (15, 16). Collagen proteins within the TME of solid cancers have recently emerged as a modulator of NK and T cell effector function (17, 18). We have shown that subcutaneous melanoma cells deposit collagen, which can inhibit tumor-recruited NK cells, and inhibiting collagen deposition turns melanoma sensitive to NK cells surveillance (17). On the contrary, NK cells can effectively control the metastasis of the subcutaneous melanoma cells to the tumor-draining lymph nodes (17). Studies reporting the accumulation of cytotoxic NK cells in the tumor-draining lymph nodes in melanoma patients suggest that NK cells can kill the disseminated tumor cells in the lymph nodes (19, 20). Likewise, we and others have utilized metastatic models to demonstrate that NK cells are critical for curbing the spread of intravenously injected cancer cells to distant organs (17, 21). NK cells may eliminate the disseminated tumor cells in the circulation as they attempt to spread to distant sites. This is supported by studies showing how clustering and platelet aggregation protect the disseminated cancer cells from NK cell-mediated killing in the circulation (22, 23).

Despite the widely appreciated function of NK cells in shaping the metastatic spread of cancers, the dynamics of NK cell-mediated control of cancer metastasis remains unclear. In this study, we utilized the B16 metastatic melanoma model to address how NK cells cooperate with T cells to reduce the metastatic tumor burden in the lungs.

## Materials and methods

### Study approval

Animal studies were approved by Massachusetts General Hospital Institutional Animal Care and Use Committee (IACUC).

### Mice

C57BL/6 wild-type (WT) mice (Charles River, Wilmington, MA, USA, strain code: 207) and C57BL/6 Ncr1^iCre^,ROSA^mT-mG^ (bred in-house) were used in the tumor experiments. All mice were housed under specific pathogen-free conditions, given water and food ad libitum, in the animal facility at Massachusetts General Hospital in accordance with animal care regulations. All mice were closely monitored by the authors, facility technicians, and an independent veterinarian when necessary. All procedures were performed according to the protocols approved by the Institutional Animal Care and Use Committee (IACUC) at Massachusetts General Hospital.

### Cell lines

WT and B2m^-/-^ B16-F10 (B16) cell lines were maintained in RPMI 1640 (Life Technologies, catalog no. 21870076) media supplemented with 10% FBS and 1% P/S and cultured at 37°C/5% CO_2_.

### Melanoma experiments

All metastatic melanoma studies were performed in eight to twelve-week-old C57BL/6 female WT or Ncr1^iCre^,ROSA^mT-mG^ mice. Mice were intravenously injected with 2x10^5^ WT or B2m^-/-^ B16 cells (in RPMI medium) *via* the tail vein or retroorbital vein. Control mice were injected with an equivalent volume of RPMI medium *via* the tail vein. Mice were euthanized 14 days post-tumor injection to collect the organs. For NK and T cell depletion, mice were intraperitoneally (I.P.) injected with 500 µg/mouse of IgG isotype control (Southern Biotech, Birmingham, AL, USA, catalog no. 0107-01), depleting anti-NK1.1 (clone PK136, BioXcell, West Lebanon, NH, USA, catalog no. BE0036), or depleting anti-CD4 (clone GK1.5, BioXcell, catalog no. BE0003-1) plus depleting anti-CD8 (clone YTS 169.4, BioXcell, catalog no. BE0117) antibodies two days before the tumor cell injection and 250 µg/mouse every other day starting the day of the tumor cell injection.

### Tissue harvesting and processing

Mice were anesthetized using ketamine/xylazine and 1 µg of monoclonal CD45-BV605 (clone 30-F11, BioLegend, San Diego, CA, catalog no. 103155) was injected by retroorbital injection for three minutes to label circulating CD45^+^ cells. Peripheral blood was collected by retroorbital bleeding and, following euthanasia, lungs and liver were collected for further processing and analysis. Red blood cells in peripheral blood were lysed using red blood cell lysis buffer (RBC lysis buffer 10X, Biolegend, catalog no. 420301). After washing with 1x DPBS/2% FCS/5 mM EDTA, 5x10^6^ cells were prepared for flow cytometry staining.

For paraffin embedding, lungs and livers were fixed in 4% Paraformaldehyde overnight at 4°C (PFA, Sigma Aldrich, catalog no. P6148) and collected for histological analysis. Subsequently, tissues were dehydrated in ethanol, processed, and embedded in paraffin according to standard histology processes. For paraffin embedding of Ncr1^icre^,Rosa^mT-mG^, a different fixation protocol was used to maintain the NKp46-GFP and Tdt-Tomato-ROSA fluorescence. Lungs from Ncr1^icre^,Rosa^mT-mG^ mice were collected and fixed in pre-chilled 95% ethanol overnight at 4°C. Tissues were subsequently dehydrated in 100% ethanol, cleared in 100% xylene, and embedded in paraffin according to standard histology processes. Blinded quantification of the metastatic lung foci was performed on macroscopic images and the size of metastatic foci was measured using the ImageJ software.

For flow analysis, lungs were chopped with scissors into ~1 mm pieces and were incubated into a 15 mL tube with 10 mL digestion buffer (RPMI 1640 (Life Technologies, catalog no. 21870076), 200 U/mL Collagenase IV (Worthington Biochemical, Lakewood, NJ, USA)) for 2 hours at 37°C with shaking. Liver lobes were collected carefully from the peritoneum of the mice without taking the gall bladder. Digested lungs and liver lobes were mashed with a plunger through the 70 µM strainer which was washed with 1x DPBS/2% FCS/5 mM EDTA by centrifuging at 300 g for 5 minutes at 4°C. Following centrifugation and aspiration of supernatant, the lungs and liver cell pellets were resuspended in 4ml 40% Percoll (Healthcare Biosciences, Uppsala, Sweden, catalog no. 17-0891-01) and gently load onto 2.5ml 70% Percoll. Samples were centrifuged at 2400 rpm for 20 minutes at 4°C without brakes and leukocytes were separated. Following washing the leukocytes with 1x DPBS/2% FCS/5 mM EDTA, the pellet was resuspended in 1x DPBS/2% FCS/5 mM EDTA buffer for flow cytometry staining.

### Flow cytometry

Single-cell suspensions from all samples were prepared by straining through a 70 µm filter. Cells were pre-incubated for 5 minutes at room temperature with CD16/CD32 masking antibody (clone 2.4G2, Tonbo Bioscience, San Diego, CA, catalog no. 70-0161-U500) to prevent non-specific antibody binding. Cells were stained with Zombie-NIR™ fixable viability dye (Biolegend, catalog no. 423106) to differentiate live/dead cells. Then, cells were stained in 1x DPBS/2% FCS/5 mM EDTA with the appropriate surface antibodies (**Table S1**) for 30 minutes at 4^°^C, washed, and analyzed by flow cytometry. For intracellular staining, cells were fixed and permeabilized using a True-Nuclear™ transcription factor kit (Biolegend, catalog no. 424401) according to the manufacturer’s protocol. Fixed cells were stained in 1x permeabilization buffer with the appropriate intracellular antibodies (**Table S1**) for 60 minutes at room temperature, washed, and analyzed by flow cytometry within a week. Cells were assayed on a BD LSR Fortessa X-20 flow cytometer (BD Bioscience, Billerica, MA, USA), and data were analyzed using FlowJo software Version 10 (Tree Star, Ashland, OR, USA). NK, CD4^+^, and CD8^+^ T cells were identified as CD3^-^NK1.1^+^NKp46^+^, NK1.1^-^CD3^+^CD4^+^, NK1.1^-^CD3^+^CD8^+^, respectively.

### Immunofluorescence staining

For IF of paraffin-embedded tissues, sections of 5 µm were rehydrated and permeabilized with 1x DPBS supplemented with 0.2% Triton X-100 (Thermo Fisher Scientific, catalog no. BP151) for 5 minutes. Antigen retrieval was then performed using a Cuisinart pressure cooker for 20 minutes at high pressure in an antigen unmasking solution (Vector Laboratories, Burlingame, CA, catalog no. H-3300). Slides were then washed three times for three minutes each in 1x DPBS supplemented with 0.1% Tween 20 (Sigma-Aldrich, catalog no. P1379). Sections were blocked, stained, and mounted as described above. Tissue sections were stained overnight at 4°C with primary antibodies (**Table S1**). The following day, slides were washed as above and incubated for two hours at RT with secondary antibodies conjugated to fluorochromes (**Table S1**). After washing as above, sections were incubated with a 1:4000 dilution of DAPI (Invitrogen, Carlsbad, CA, catalog no. D3571) in 1x TBS for 5 minutes at RT, washed, air dried and coverslips mounted with Prolong Gold Antifade Reagent (Invitrogen, catalog no. P36930). Five or six randomly selected fields of view at 200x total magnification were obtained for each section using a Zeiss Axio Scan (Zeiss, Oberkochen, Germany). Blinded manual counting of NKp46^+^, CD4^+^, and CD8^+^ T cells was performed using ZEN Blue Software (Zeiss, Oberkochen, Germany). Whole slide imaging was performed using a NanoZoomer S60 Digital slide scanner (Hamamatsu, Japan) and analyzed with NDP-view2 software (Hamamatsu).

### Statistical analysis

Bar graphs show mean values + standard deviation (SD). The numbers of mice per group used in each experiment are annotated in the corresponding figure legend as *n.* Graphs and statistical analysis were performed using GraphPad Prism 8 (La Jolla, CA, USA). All tumor quantifications were performed blindly. One-way ANOVA and Two-tailed Mann-Whitney *U* tests were used for the comparisons. A *P* value of less than 0.05 was considered significant.

## Results

### NK and T cells play important but distinct roles in the control of melanoma metastasis

**Figure 1:**
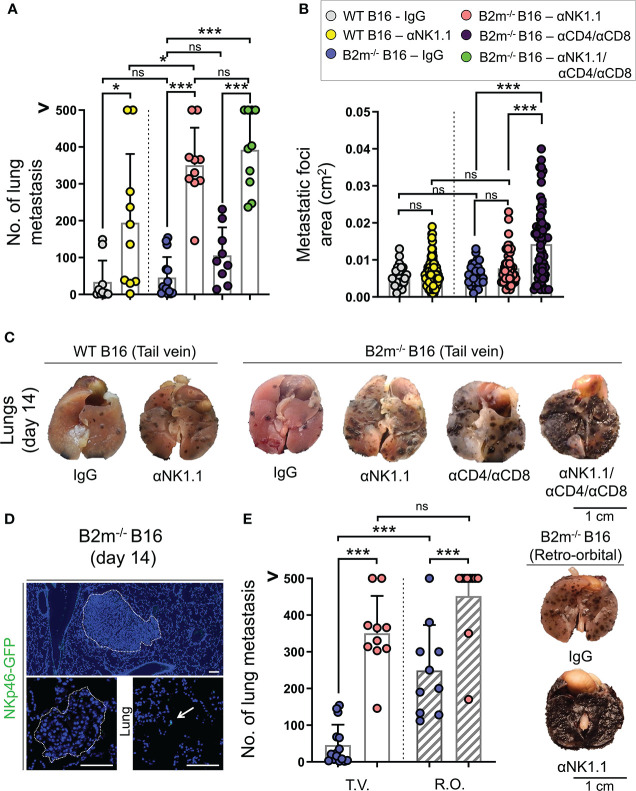
Distinct roles of NK and T cells in controlling the pulmonary metastasis of B16 melanoma. **(A)** Quantification of WT and B2m^-/-^ B16 melanoma metastatic foci in the lung following tail vein IV melanoma cell injection and treatment with control IgG, anti-NK1.1, anti-CD4/CD8, anti-NK1.1/CD4/CD8 antibodies. n = 9-15 mice per group. **(B)** Quantification of the area of WT and B2m^-/-^ B16 melanoma metastatic foci in the lungs following tail vein IV melanoma cell injection and treatment with control IgG, anti-NK1.1, anti-CD4/CD8, anti-NK1.1/CD4/CD8 antibodies. n = 27-87 macroscopic metastatic foci measured per group. **(C)** Representative macroscopic images of lung metastases of WT and B2m_-/-_ B16 melanoma at day 14 post-tail vein IV injection of melanoma cells. Antibody treatments are listed below the lung images. **(D)** Representative IF images of large and small B2m^-/-^ B16 melanoma lung metastases in Ncr1^iCre^,ROSA^mT-mG^ reporter mice at day 14 post-tail vein IV melanoma cell injection. The presence of NKp46-GFP+ NK cells (green) in the lung is highlighted with white arrow. Dash lines outline the melanoma metastatic foci in the lung. **(E)** Quantification of B2m^-/-^ B16 melanoma metastatic foci in the lung following tail vein (T.V.) and retro-orbital (R.O.) IV melanoma cell injection and treatment with control IgG and anti-NK1.1 antibody. n = 10-15 mice per group. Note that the data for T.V. groups are also shown in **(A)**. **(F)** Representative macroscopic images of lung metastases of B2m^-/-^ B16 melanoma at day 14 post-retro-orbital IV melanoma cell injection. Antibody treatments are listed below lung images. **(A, B, E)** Graphs show mean + SD, one-way ANOVA, ns: not significant, * *p* < 0.05, *** *p* < 0.001, **(C, F)** Scale bars = 1 cm, **(D)** Scale bars = 100 µm.

To investigate the role of NK and T cells in the surveillance of cancer metastasis, we employed the B16 metastatic melanoma model together with antibody-mediated depletion of NK and T cells. B16 melanoma cell line with endogenous expression of MHC-I (WT B16) and with loss of MHC-I through *B2m* gene deletion (B2m^-/-^ B16) were utilized. Removal of MHC-I, a ligand for inhibitory Ly49 receptors on mouse NK cells, is an established approach to induce NK cell activation (24). However, loss of MHC-I did not significantly alter number or size of metastatic foci in the lungs, which may be because B16 cells endogenously express very low levels of MHC-I (**Figures 1A–C**, **Figure S1**) (25).

To identify the anti-tumor role of NK and T cells, mice were treated with NK and CD4/CD8 T cell-depleting antibodies to eliminate NK and T cells in the metastatic melanoma model, respectively. For control, mice were treated with IgG isotype antibody, which did not affect NK and T cells. Compared to IgG isotype-treated mice, NK cell depletion resulted in a significant increase in the numbers of metastatic foci in the lungs on day 14 following tail vein injections of WT and B2m^-/-^ B16 cells (**Figures 1A** **, C**). Surprisingly, NK cell depletion did not alter the size of WT and B2m^-/-^ B16 metastatic foci in the lungs compared to the IgG treatment (**Figures 1B, C**). In contrast, the depletion of CD4^+^ and CD8^+^ T cells did not affect the number of B2m^-/-^ B16 lung metastasis but significantly increased the size of metastatic foci compared to IgG treatment (**Figures 1A** **–C**). These findings indicate that NK cells are required to block the seeding of metastatic B16 melanoma cells in the lung, while T cells are not prominent players in this process but rather control the growth of the metastatic foci formed in the lung. Interestingly, there was a slight but significant increase in the number of lung metastasis of B2m^-/-^ B16 compared to that of WT B16 melanoma cells in NK cell-depleted groups, indicating that T cells may utilize the MHC-I axis to play a minor compensatory role in limiting the number of lung metastasis in the absence of NK cells (**Figures 1A, C**).

Consistently, the depletion of NK plus T cells worsened the metastatic burden of B2m^-/-^ B16 melanoma cells in the lungs compared to the NK cell depletion, which likely indicates the cooperative role of NK cell-mediated restraint of B16 lung seeding and T cell-mediated control of metastatic foci growth in the lungs (**Figure 1A**). Notably, the combined depletion of NK and T cells resulted in an increased number of B2m^-/-^ B16 metastatic foci that were large and fused on day 14, preventing the accurate quantification of each metastatic focus (**Figure 1C**).

Despite the pivotal role of NK cells in limiting the B16 melanoma lung metastasis, we did not detect NK cells infiltrating the metastatic foci in the lungs. However, there were infrequent NK cells within the parenchyma of the lung away from the metastatic foci on day 14 post-tail vein cancer cell injection (**Figure 1D**). This finding suggested that NK cells cleared the metastatic cancer cells outside the lung to prevent their pulmonary seeding. To examine whether NK cell clearance of melanoma cells took place in the circulation, we changed the route of melanoma cell delivery from the tail vein to retroorbital vein injection to avoid the retrograde blood flow in the liver (26). Interestingly, the retroorbital vein injection of B2m^-/-^ B16 melanoma cells led to a massive increase in lung metastasis (**Figures 1E, F**). This finding strongly suggests that the liver is a prominent site where productive interactions between NK cells and metastatic cancer cells occur to control pulmonary melanoma cell seeding. Accordingly, occasional liver metastasis of intravenously injected B2m^-/-^ B16 cells was noted only in the mice depleted of NK cells. In addition, the combined depletion of NK plus T cells in the mice that received B2m^-/-^ B16 cells *via* tail vein injection led to the emergence of extrapulmonary metastasis in various organs such as the liver, kidney, stomach, ovaries, and uterine horns (**Figure S2**). These findings further highlight the cooperative role of NK and T cells in controlling extrapulmonary metastasis of melanoma in various organs.

### NK cells regulate the infiltration of T cells into the metastatic foci in the lung

**Figure 2:**
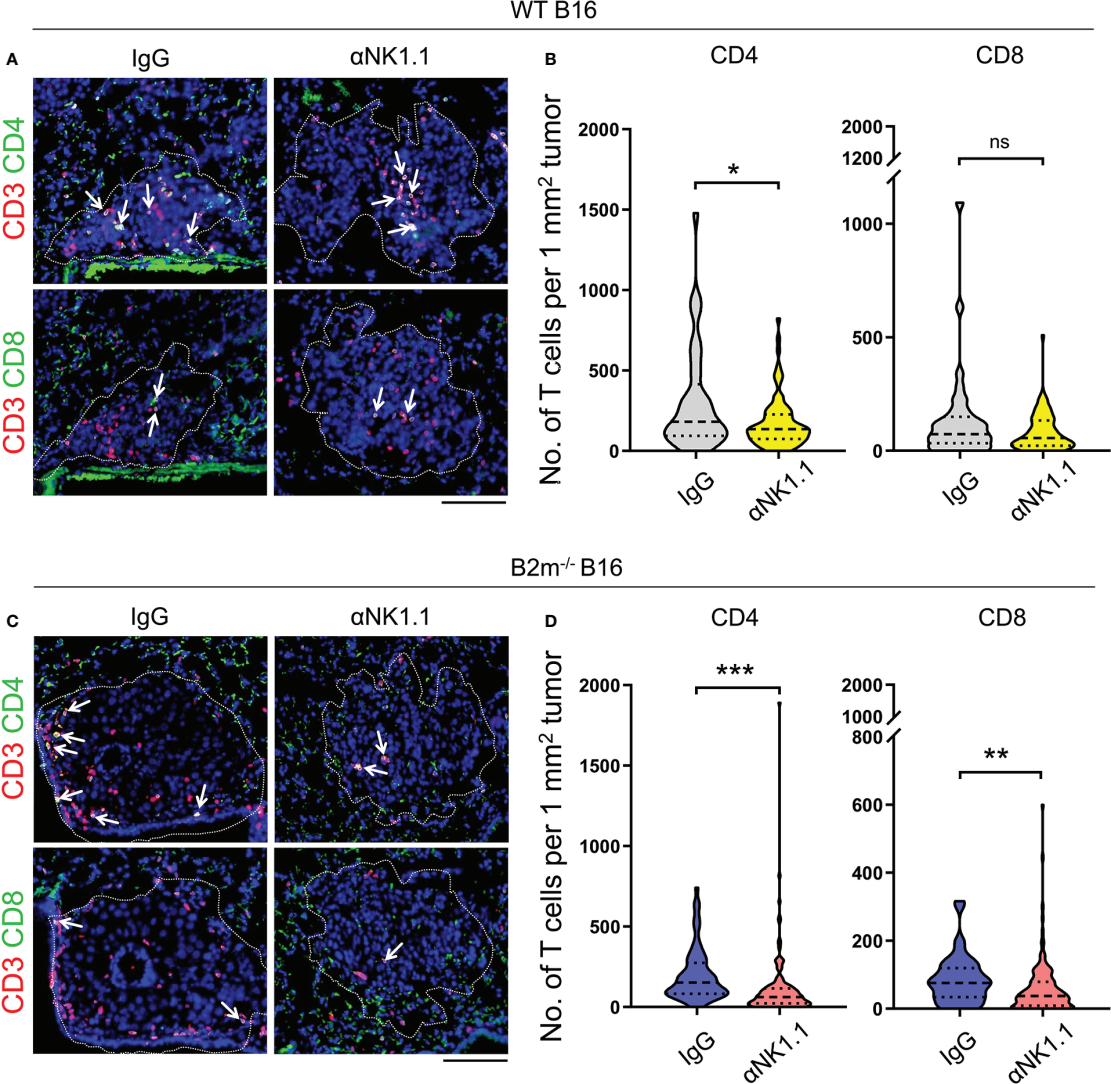
NK cells control T cell recruitment to melanoma foci in the lungs. **(A, B)** Representative images **(A)** and quantification **(B)** of CD4^+^ T cells (CD4/CD3 stained) and CD8^+^ T cells (CD8/CD3 stained) in WT B16 melanoma lung metastases. **(C, D)** Representative images **(C)** and quantification **(D)** of CD4^+^ T cells (CD4/CD3 stained) and CD8^+^ T cells (CD8/CD3 stained) in B2m^-/-^ B16 melanoma lung metastases. Antibody treatments are listed above IF images and below the quantification graphs. **(A, C)** Dash lines outline the melanoma metastatic foci in the lung where T cells are highlighted with white arrows. Scale bars = 100 μm. **(B, D)** T cells were counted in 35-140 metastatic foci between 0.01 to 0.2 mm^2^ per group. Mann-Whitney *U* test, ns, not significant, *p < 0.05, **p < 0.01, ***p < 0.001.

Based on the pivotal role of T cells in curbing melanoma metastatic foci growth in the lungs, we assessed the infiltration of CD4^+^ and CD8^+^ T cells in the lungs on day 14 following the tail vein injection of WT and B2m^-/-^ B16 melanoma. Although the metastatic lung foci at the endpoint were largely devoid of NK cells, we found prominent infiltration of CD4^+^ and CD8^+^ T cells into the WT B16 metastatic foci in the lungs (**Figures 2A, B**). NK cell depletion significantly reduced T cell infiltration into WT B16 metastatic foci (**Figures 2A, B**). Although CD4^+^ and CD8^+^ T cells had lower infiltration into B2m^-/-^ B16 metastatic foci compared to WT B16 metastatic foci, they infiltrated B2m^-/-^ B16 metastatic foci in an NK cell-dependent manner (**Figures 2C, D**). These findings demonstrate that NK cells, despite their absence in the metastatic foci, play a prominent role in promoting T cell recruitment into the metastatic foci in the lung.

### NK and T cells increase in the liver and lung, respectively, in response to metastatic melanoma

**Figure 3:**
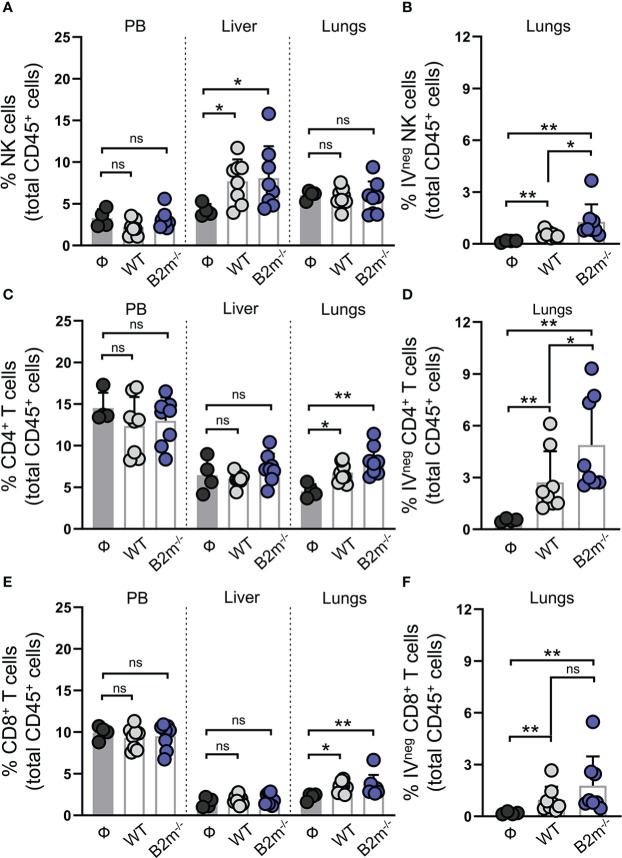
NK cells are enriched in the liver while T cells are enriched in the lung in response to metastatic melanoma. **(A)** Quantification of total NK1.1^+^ NKp46^+^ NK cells in peripheral blood (PB), liver and lungs at day 14 after WT B16 (WT) and B2m^-/-^ B16 (B2m^-/-^) melanoma tail vein IV injection in comparison with untreated mice (Ф). **(B)** Quantification of lung-infiltrating IV^neg^ NK cells at day 14 after WT B16 (WT) and B2m^-/-^ B16 (B2m^-/-^) melanoma tail vein IV injection in comparison with untreated mice (Ф). **(C)** Quantification of total CD4^+^ T cells in PB, liver and lungs at day 14 after WT B16 (WT) and B2m^-/-^ B16 (B2m^-/-^) melanoma tail vein IV injection in comparison with untreated mice (Ф). **(D)** Quantification of lung-infiltrating IV^neg^ CD4^+^ T cells at day 14 after WT B16 (WT) and B2m^-/-^ B16 (B2m^-/-^) melanoma tail vein IV injection in comparison with untreated mice (Ф). **(E)** Quantification of total CD8^+^ T cells in PB, liver and lungs at day 14 after WT B16 (WT) and B2m^-/-^ B16 (B2m^-/-^) melanoma tail vein IV injection in comparison with untreated mice (Ф). **(F)** Quantification of lung-infiltrating IV^neg^ CD8^+^ T cells at day 14 after WT B16 (WT) and B2m^-/-^ B16 (B2m^-/-^) melanoma tail vein IV injection in comparison with untreated mice (Ф). n = 4-8 mice per group. Graphs show mean + SD. Mann-Whitney *U* test, ns, not significant, *p < 0.05, **p < 0.01.

We next characterized NK and T cells in mice on day 14 post-tail vein injection of WT and B2m^-/-^ B16 melanoma cells. Immune cells from peripheral blood (PB), lungs, and liver were collected to quantify NK, CD4^+^, and CD8^+^ T cells proportions among total CD45^+^ immune cells in the organs (**Figure S3A**). For specific evaluation of the immune cells infiltrating into the lung tissue, fluorescently labeled anti-CD45 antibody was intravenously administered to mice three minutes before organ harvest. This enabled efficient tagging of all the immune cells in the vasculature while the tissue-resident/emigrated immune cells remained unlabeled (17). CD45-stained immune cells from the vasculature are termed CD45 IV^+^ (IV stands for intravenous) while tissue-resident/emigrated immune cells which remained unstained after intravenously injection of CD45 antibody are termed CD45 IV^-^. Following the organ harvest, immune cells were stained *ex vivo* by distinctly labeled CD45 antibody to stain all immune cells. As expected, 100% of the *ex vivo* CD45-stained immune cells from peripheral blood (PB) were found to be positive for intravenously injected CD45 antibody (CD45 IV^+^), while a proportion of *ex vivo* CD45-stained lung immune cells were negative for intravenously injected CD45 antibody (CD45 IV^-^) (**Figure S3B**). Compared to the mice that were not injected with cancer cells (Ф), WT and B2m^-/-^ B16 melanoma cell administration through the tail vein led to a significant increase in the proportions of NK cells in the liver (**Figure 3A**). The percentage of NK cells among total CD45^+^ immune cells in PB and lungs remained unaltered between melanoma cell-injected and control mice (**Figure 3A**). There was no significant difference in the NK cell proportions in the organs between mice that received WT versus B2m^-/-^ B16 melanoma cells (**Figure 3A**). Quantifying IV^neg^ NK cells in the lungs revealed a slight but significant enrichment of lung-infiltrating NK cells in WT B16 melanoma cell-injected mice, which further increased in mice injected with B2m^-/-^ B16 melanoma cells (**Figure 3B**). On the other hand, T cell quantification showed significant enrichment of total and IV^neg^ proportions of CD4^+^ and CD8^+^ T cells in the lungs of mice that had received WT and B2m^-/-^ B16 tumors (**Figures 3C** **–F**). In contrast, T cell proportions were not affected by melanoma cells in the liver (**Figures 3C, E**). Of note, among IV^neg^ immune effector populations in the lungs, T cells, especially CD4^+^ T cells, showed the most robust increase in response to WT and B2m^-/-^ B16 melanoma challenge compared to the untreated control mice (**Figures 3B, D, F**). Thus, these data strongly indicate that tumor burden in the lungs promotes modest recruitment of circulatory NK cells to the lungs, which are largely excluded from the metastatic foci in the lungs. Although NK cells lack a direct ability to curb the growth of metastatic foci in the lungs, they regulate CD4^+^ and CD8^+^ T cell recruitment to the lungs, which infiltrate and control the *in situ* metastatic tumor growth in the lungs.

### Activated circulatory NK cells are recruited to the lungs in response to the metastatic melanoma

**Figure 4:**
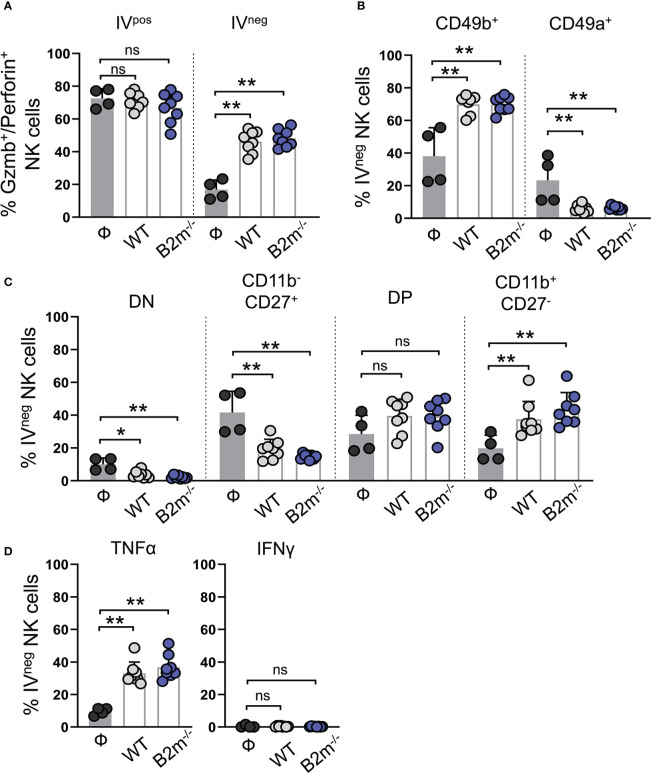
Characterization of lung-infiltrating NK cells in response to metastatic melanoma. **(A)** Quantification of granzyme B^+^/perforin^+^ circulating (IV^pos^) and lung-infiltrating (IV^neg^) NK cells at day 14 after WT B16 (WT) and B2m^-/-^ B16 (B2m^-/-^) melanoma tail vein IV injection in comparison with untreated mice (Ф). **(B)** Quantification of conventional (CD49b^+^) and tissue-resident (CD49a^+^) lung-infiltrating (IV^neg^) NK cells at day 14 after WT B16 (WT) and B2m^-/-^ B16 (B2m^-/-^) melanoma tail vein IV injection in comparison with untreated mice (Ф). **(C)** Quantification of maturation status defined by CD11b and CD27 expression in lung-infiltrating (IV^neg^) NK cells at day 14 after WT B16 (WT) and B2m^-/-^ B16 (B2m^-/-^) melanoma tail vein IV injection in comparison with untreated mice (Ф). **(D)** Quantification of TNFα^+^ and IFNγ^+^ lung-infiltrating (IV^neg^) NK cells at day 14 after WT B16 (WT) and B2m^-/-^ B16 (B2m^-/-^) melanoma tail vein IV injection in comparison with untreated mice (Ф). n = 4-8 mice per group. Graphs show mean + SD. Mann-Whitney *U* test, ns, not significant, *p < 0.05, **p < 0.01.

Next, we assessed lung-recruited NK and T cells on day 14 post-tail vein injection of WT and B2m^-/-^ B16 melanoma cells. Intravenous administration of fluorescently labeled anti-CD45 antibody allowed us to differentiate the lung-resident/recruited IV^neg^ NK cells from those in the lung vasculature marked as IV^pos^ (**Figure S4A**). IV^neg^ and IV^pos^ NK cells were quantified for the expression of functional markers (**Figures S4B–E**). Likewise, IV^neg^ and IV^pos^ CD3^+^ T cells were separated by CD45 IV labeling followed by gating for CD4^+^ and CD8^+^ T cells and quantification of functional markers (**Figures S4F–L**). In tumor-free mice, most IV^pos^ NK cells in the circulation were positive for granzyme B and perforin, while only a minor fraction of IV^neg^ NK cells in the tumor-free lungs showed granzyme B/perforin expression (**Figure 4A**). Intravenous WT and B2m^-/-^ B16 melanoma challenge resulted in a significant increase in the proportions of granzyme B/perforin positive IV^neg^ NK cells in the lungs (**Figure 4A**). Tissue residency of NK cells was defined by the mutually exclusive expression of integrins CD49b versus CD49a (27). The majority of IV^pos^ circulating NK cells in the lung were CD49b^+^CD49a^-^ conventional NK (cNK) cells in WT, B2m^-/-^ B16 melanoma-challenged and untreated mice (**Figure S5A**). In contrast, among IV^neg^ lung-infiltrating NK cells, we found significantly increased cNK cells and decreased CD49b^-^CD49a^+^ tissue-resident NK (trNK) cells proportions upon WT and B2m^-/-^ B16 melanoma challenge (**Figure 4B**).

The relative expression and surface density of integrin CD11b (Mac-1) and CD27 distinguish NK cells in four different maturation and functional stages. CD11b^-^CD27^-^ (double negative, DN) and CD11b^-^CD27^+^ NK cells are closely related and developmentally immature precursor-like NK cells that give rise to more mature and cytotoxic CD11b^+^CD27^+^ (double positive, DP) and CD11b^+^CD27^-^ populations (28). In control mice, almost all IV^pos^ NK cells in the lungs were mature and functionally potent NK cells with DP and CD11b^+^CD27^-^ phenotype (**Figure S5B**). Tail vein injections of WT and B2m^-/-^ B16 melanoma cells did not alter the CD11b^+^ mature NK cell dominance, but it significantly decreased IV^pos^ DP population and likely led to their conversion into CD11b^+^CD27^-^ NK cells in the circulation (**Figure S5B**). In the absence of WT or B2m^-/-^ B16 melanoma cells, almost 50% of IV^neg^ NK cells in the lungs showed an immature precursor-like phenotype with a lack of CD11b marker expression (**Figure 4C**). WT and B2m^-/-^ B16 melanoma challenge significantly reduced the IV^neg^ proportions of DN and CD11b^-^CD27^+^ precursors and increased functionally dynamic and mature NK cell populations comprising DP and CD11b^+^CD27^-^ NK cells (**Figure 4C**). TNFα expression in IV^pos^ circulating and IV^neg^ lung-infiltrating NK cells was significantly enhanced in the lungs of mice that received WT and B2m^-/-^ B16 melanoma cells (**Figures 4D**, **S5C**). On the other hand, IFNγ expression was undetectable irrespective of the tumor challenge in both IV^pos^ and IV^neg^ NK cell populations in the lungs (**Figures 4D**, **S5C**). In addition to circulating NK cells, liver NK cells in mice that received WT and B2m^-/-^ B16 melanoma were activated compared to the control mice on day 14-post melanoma challenge (**Figures S5D, E**).

### T cells are activated *in situ* in the lungs in response to the metastatic melanoma

**Figure 5:**
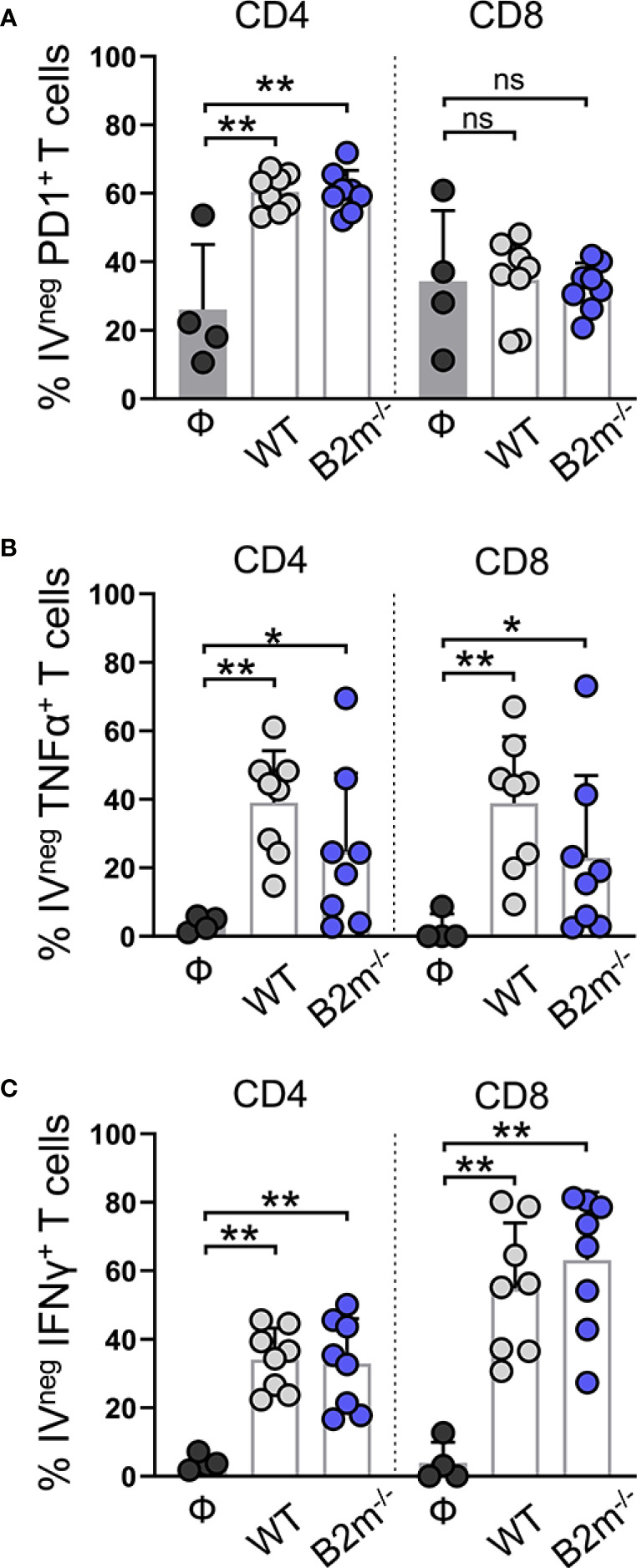
Characterization of lung-infiltrating T cells in response to metastatic melanoma. **(A–C)** Quantification of PD1^+^ **(A)**, TNFα^+^ **(B)**, and IFNγ^+^ **(C)** lung-infiltrating (IV^neg^) CD4^+^ and CD8^+^ T cells at day 14 after WT B16 (WT) and B2m^-/-^ B16 (B2m^-/-^) melanoma tail vein IV injection in comparison with untreated mice (Ф). n = 4-8 mice per group. Graphs show mean + SD. Mann-Whitney *U* test, ns, not significant, *p < 0.05, **p < 0.01.

We found that PD1^+^ IV^pos^ CD4^+^ T cells remained comparable between the control and WT and B2m^-/-^ B16 melanoma-challenged mice (**Figure S6A**, “CD4”). In contrast, PD1 expression on IV^neg^ CD4^+^ T cells from the lungs was significantly elevated in WT and B2m^-/-^ B16 melanoma-challenged mice (**Figure 5A**, “CD4”). In WT and B2m^-/-^ B16 tumor-bearing lungs, around 40-50% IV^neg^ CD8^+^ T cells expressed PD1 compared to only 10-20% IV^pos^ CD8^+^ T cells in the circulation (**Figures 5A**, **S6A**, “CD8”). Upon lung infiltration, IV^neg^ CD4^+^ and CD8^+^ T cells significantly upregulated TNFα expression in WT and B2m^-/-^ B16 melanoma-challenged mice compared to the controls (**Figure 5B**). In circulation, only IV^pos^ CD8^+^ T cells from mice that received WT B16 melanoma cells significantly upregulated TNFα expression (**Figure S6B**). In contrast to NK cells, IV^pos^ and IV^neg^ T cells showed robust expression of IFNγ in the lungs of WT and B2m^-/-^ B16 melanoma-challenged mice compared to control animals (**Figures 5C**, **S6C**).

## Discussion

Our findings demonstrate the cooperative function of NK and T cells in the surveillance of metastatic melanoma. Our data strongly indicate that NK cells and T cells are performing complementary roles in controlling B16 melanoma cells, which dominantly metastasize to the lungs (29). While NK cells drastically reduce the number of metastatic foci in the lungs, they essentially do so by clearing the metastatic cancer cells in circulation. Although NK cells from the melanoma-bearing lungs are largely excluded from the established B16 metastatic foci, they promote the recruitment and infiltration of CD4^+^ and CD8^+^ T cells into the metastatic foci in the lungs. In turn, tumor-infiltrating T cells show robust *in situ* activation in melanoma-challenged mice and play a pivotal role in curbing the growth of the metastatic foci formed in the lungs. Thus, strategies to boost NK cells recruitment and cytotoxicity in the metastatic foci in the lungs can further enhance antitumor T cell activation and lead to optimal suppression of the metastatic disease.

Our study demonstrates the critical role of NK cells in limiting the metastatic melanoma burden in the lungs despite their lack of infiltration into the established metastatic foci. This indicates that NK cell-mediated metastatic tumor surveillance occurs outside the lung tissue. Cancers in the lung generally lack a robust NK cell infiltration, more commonly seen during various pulmonary infections where NK cells kill the infected cells and promote antigen-specific adaptive immune response (30). In one study, the authors employed two different methods to deliver a Lewis lung carcinoma (3LL) cell line to the lung to assess NK cell response (31). While orthotopic implantation of 3LL led to the recruitment of migratory NK cells in tumor-bearing lungs in a CXCR3-dependent manner, the control of lung-inoculated 3LL cells was mediated by lung-resident NK cells (31). On the other hand, pulmonary metastasis of intravenously administered 3LL cells was significantly reduced by NK cells, albeit in a CXCR3-independent manner, suggesting that the recruitment of migratory NK cells into the lungs is not required to inhibit lung metastasis of metastatic 3LL cells (31).

Lung is a highly vascularized organ that may enable effective immune surveillance of cancer within the pulmonary vasculature. Our findings demonstrate that large numbers of NK cells in the lung capillaries presented highly cytotoxic and mature phenotypes at baseline, which remained unaltered upon tumor challenge. Conceivably, the lethal encounters between the NK cells and circulatory melanoma cells within the lung capillaries reduced the number of metastatic foci in the lung parenchyma. This is corroborated by a recent study that utilized bioluminescence imaging with intravital two-photon microscopy to unravel early immunosurveillance of metastatic melanoma cells (32). The authors find that most productive interactions between NK cells and intravenously injected B16 melanoma cells occur within the pulmonary capillary bed during the first 12 hours after tail vein injection. Further, it is observed that disseminated tumor cells start to form NK cell-resistant macrometastatic nodules in the lung parenchyma as early as 24 hours after the injection (32).

Interestingly, our findings reveal no macroscopic B16 metastatic foci in the liver unless NK cells are depleted, which indicates that NK cells can effectively control the B16 metastatic burden in the liver. Indeed, enriched NK cells in the livers at the study endpoint imply an active NK cell surveillance of microscopic B16 nodules in the liver. In addition, we postulate that liver NK cells may also regulate the pulmonary seeding of tail vein-administered tumor cells. We have found a significantly increased number of metastatic melanoma foci in the lungs upon changing the route of intravenous administration *via* the retroorbital vein, thereby avoiding the retrograde blood flow in the liver (33). It is plausible that retroorbital delivery prevented some of the productive NK-tumor interactions while bypassing the first liver passage resulting in higher pulmonary seeding of B16 melanoma cells. The precise role of liver NK cells in controlling liver metastasis has been recently uncovered using genetically modified mice that lack conventional NK (cNK) or tissue-resident NK (trNK) cells (34). In this study, the authors showed that intra-splenic injections of tumor cells result in liver metastasis, which is collaboratively controlled by cNK and trNK cells (34). The authors found that trNK cells restricted the hepatic seeding of the metastatic tumor cells, while the cNK cells inhibited the metastatic outgrowth in the livers (34). Further, the authors speculate that CXCR6-dependent retention of trNK cells in hepatic sinusoids likely explains their critical role in regulating tumor cell seeding (34). In light of this evidence, trNK cells in the hepatic sinusoids may have controlled the seeding of intravenously injected B16 melanoma cells to control their pulmonary seeding in our system.

Moreover, NK cell depletion further increased the metastatic burden of melanoma in the lungs following retroorbital delivery. This reaffirms the findings by Ichise et al. (32) that NK cells perpetually clear the floating tumor cells during the first few hours of circulation. Therefore some of the tumor cells that escaped NK cell killing during the first liver passage following the retroorbital injection may eventually succumb to liver NK cells during the recirculation. In the past, numerous studies have compared the tail vein and retroorbital administration routes for their influence on the kinetics of injected antibodies and traceable dyes (26, 35, 36). While these studies proved both administration routes to be identical, the delivery of tumor cells in immunocompetent mice induces a more dynamic response, likely producing divergent phenotypes in the present study.

In contrast to NK cells, we find a profound increase in lung-infiltrating T cells upon melanoma challenge. Interestingly, NK cells induce the infiltration of the T cells into the metastatic foci in the lungs. NK cell-mediated recruitment and activation of T cells are established in several disease models. Using the skin transplantation model, we have shown that CD4^+^ and CD8^+^ T cell recruitment to the skin graft depends on and follows the skin infiltration of circulating NK cells in response to mouse cytomegalovirus-encoded protein, m157 (17). The role of NK cell-derived CCL5 and XCL1 in the recruitment of conventional type 1 dendritic cells (cDC1) leading to the anti-tumor CD8^+^ T cell response is identified in multiple subcutaneous tumor models including melanoma (37). Similarly, in the lung adenocarcinoma model with inducible expression of NK cell activating ligands, NK cell infiltration into the lungs stimulated T cell recruitment and activation, leading to a heightened adaptive immune response against lung tumors (38). In this study, flow cytometry-based characterization shows that CD4^+^ and CD8^+^ T cells become activated *in situ* once they exit the circulation and enter the tumor-bearing lungs. Of note, CD8^+^ T cell activation status was similar irrespective of WT or B2m^-/-^ B16 melanoma in the lungs. WT B16 cells are deficient for surface MHC-I expression and demonstrate weak immunogenicity, which may be the reason for similar CD8^+^ T cell reactivity observed in the lungs of WT or B2m^-/-^ B16 injected mice (25). Nonetheless, a role for CD8^+^ T cells in the retardation of tumor growth in the lungs is plausible as there is a significant induction of TNFα and IFNγ in lung-infiltrating CD8^+^ T cells in the melanoma-bearing lungs. These cytokines can thwart cancer growth directly or *via* potentiation of the immune response (39). Moreover, bystander activation of CD8^+^ T cells independent of MHC-I expression is reported and may play a role in our findings (40, 41). Bystander activated CD8^+^ T cells become activated *via* NKG2D receptor to kill the NKG2D-ligand expressing target cells in the absence of productive T cell receptor (TCR) engagement by MHC-I (40, 41). In addition, CD8^+^ T cells can utilize a novel mechanism that is independent of TCR and NKG2D axis but still depends upon cell-cell contact and PI3K signaling to kill the target cells (40, 41). We also find a prominent *in situ* activation of CD4^+^ T cells in WT and B2m^-/-^ B16 tumor-bearing lungs. In addition to their well-established helper role, the direct tumoricidal activity of CD4^+^ T cells against MHC-II^+^ and MHC-II^-^ tumors are documented (42, 43). IFNγ could upregulate the expression of MHC-II on B16 cells, which augmented CD4^+^ T cell-mediated killing of melanoma (42, 43). We speculate this axis to be critical in controlling B16 metastatic outgrowth in our system.

Finally, our study identifies a unique cooperative role of NK and T cells in limiting B16 metastasis in several extrapulmonary organs such as ovaries, kidneys, uterine horns, and stomach. It has been reported that the estrous cycle status of the mice at the time of intravenous delivery of B16 cells can lead to their metastasis to the ovaries (44). This is highly unlikely in our experiments as none of the mice in other groups showed macroscopic B16 metastasis except for all the mice in the NK and T cell combined depletion group. In addition, we find melanoma metastasis in non-reproductive organs such as the kidney and stomach, which are not likely to be affected by the estrous cycle. In addition to being permissive to circulatory NK and T cells, these organs are also known to harbor tissue-resident NK and T cells. It is possible that the distinct roles of NK and T cells that we observed in controlling pulmonary metastasis may not extend to extrapulmonary organs. Especially uterine horns are NK cell-rich organs where NK cells may control the *in situ* tumor growth similar to T cells. How NK and T cells act together to control the extrapulmonary metastasis of cancer remains to be investigated in future research.

## Data availability statement

The original contributions presented in the study are included in the article/**Supplementary Material**. Further inquiries can be directed to the corresponding author.

## Ethics statement

The animal studies were approved by Massachusetts General Hospital Institutional Animal Care and Use Committee (IACUC).

## Author contributions

MV, MR and SD conceived the study. MV, MR, TN and SD designed the experiments. MV, MR, TN, DP and MA performed the experiments and analyzed the data. MV, MR, TN, DP, MA and SD interpreted the data. MV and SD wrote the manuscript. All authors contributed to the article and approved the submitted version.
